# Supplementary material for: MCC Gene Silencing Is a CpG Island Methylator Phenotype-Associated Factor That Predisposes Colon Cancer Cells to Irinotecan and Olaparib
Source: Cancers (Basel). 2022 Jun 9;14(12):2859. doi: 10.3390/cancers14122859 (PMC9221012; doi:10.3390/cancers14122859)
Supplement: Supplementary file 1 [file cancers-14-02859-s001.zip › Figure S1-3&Table S1-2.pdf]

Supplementary Figures and Tables.

Zeenat Jahan, Fahad Benthani, Nicola Currey, Hannah W Parker, Jane E Dahlstrom, C Elizabeth Caldon, Maija RJ Kohonen-Corish.
MCC gene silencing is a CpG island methylator phenotype-associated factor that predisposes colon cancer cells to irinotecan and olaparib. Cancers 2022; 14

Figure S1: Comparison of *MCC-201* mRNA expression levels with CpG site methylation beta-values in the TCGA COAD cohort.

Figure S2: Comparison of *MCC-201* mRNA expression levels with CpG site methylation beta-values in the TCGA READ cohort.

Figure S3: PARP sub-cellular localization after SN38 exposure of HCT116 cells.

Table S1: *MCC-201* mRNA expression level and CpG site methylation beta-values in the TCGA COAD cohort.

Table S2: *MCC-201* mRNA expression level and methylation beta-values in the TCGA READ cohort.

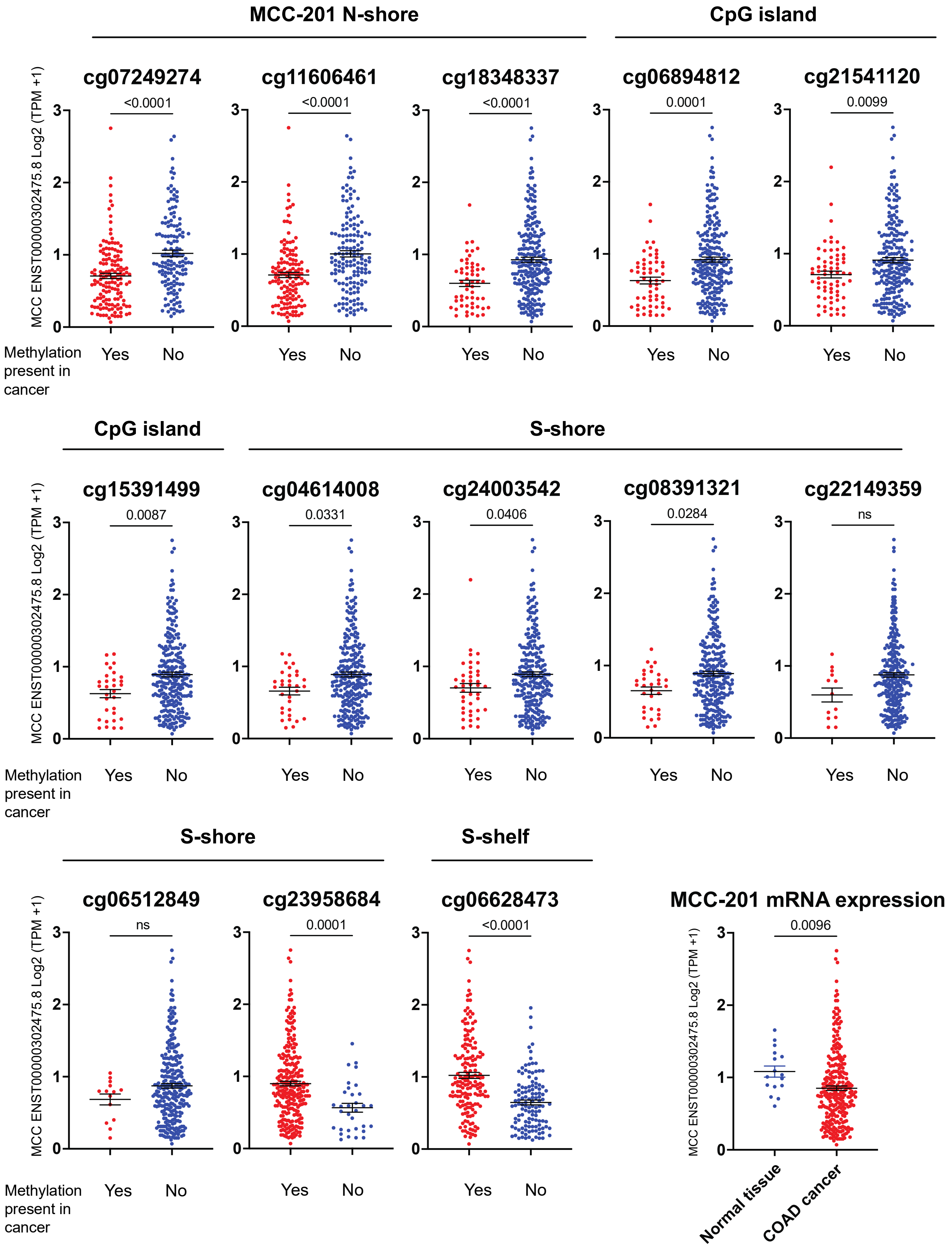

Supplementary Figure S1. Down-regulation of the *MCC*-201 transcript in colon cancer is associated with hypermethylation of individual CpG sites in the N-shore, CpG island and S-shore or hypomethylation in the S-shore/S-shelf (TCGA 2018 COAD cohort). Statistical significance was determined using the unpaired Mann-Whitney test. Error bars show mean ± SEM. Methylation beta-values >0.5 were considered as hypermethylated in cancer if the matching CpG site was unmethylated in normal tissue. Methylation beta-values <0.4 were considered as hypomethylated in cancer if the site was methylated in normal tissue. Detailed data are shown in Supplementary Table S1.

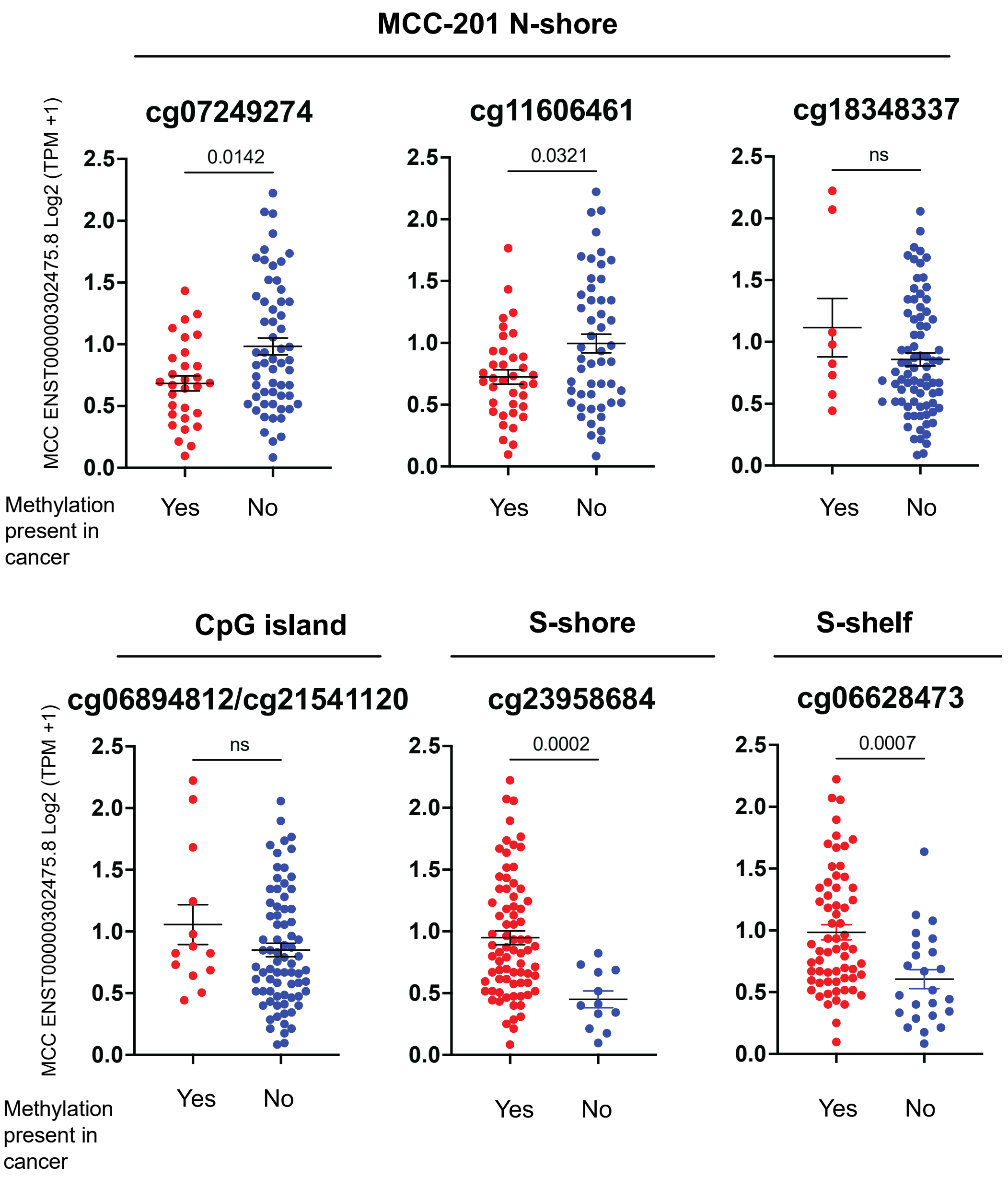

Supplementary Figure S2. Down-regulation of the *MCC*-201 transcript in rectal cancer is associated with hypermethylation of individual CpG sites in the N-shore or hypomethylation in the S-shore/S-shelf (TCGA 2018 READ cohort). Statistical significance was determined using the unpaired Mann-Whitney test. Error bars show mean ± SEM. Methylation beta-values >0.5 were considered as hypermethylated in cancer if the matching CpG site was unmethylated in normal tissue. Methylation beta-values <0.4 were considered as hypomethylated in cancer if the site was methylated in normal tissue. Detailed data are shown in Supplementary Table S2.

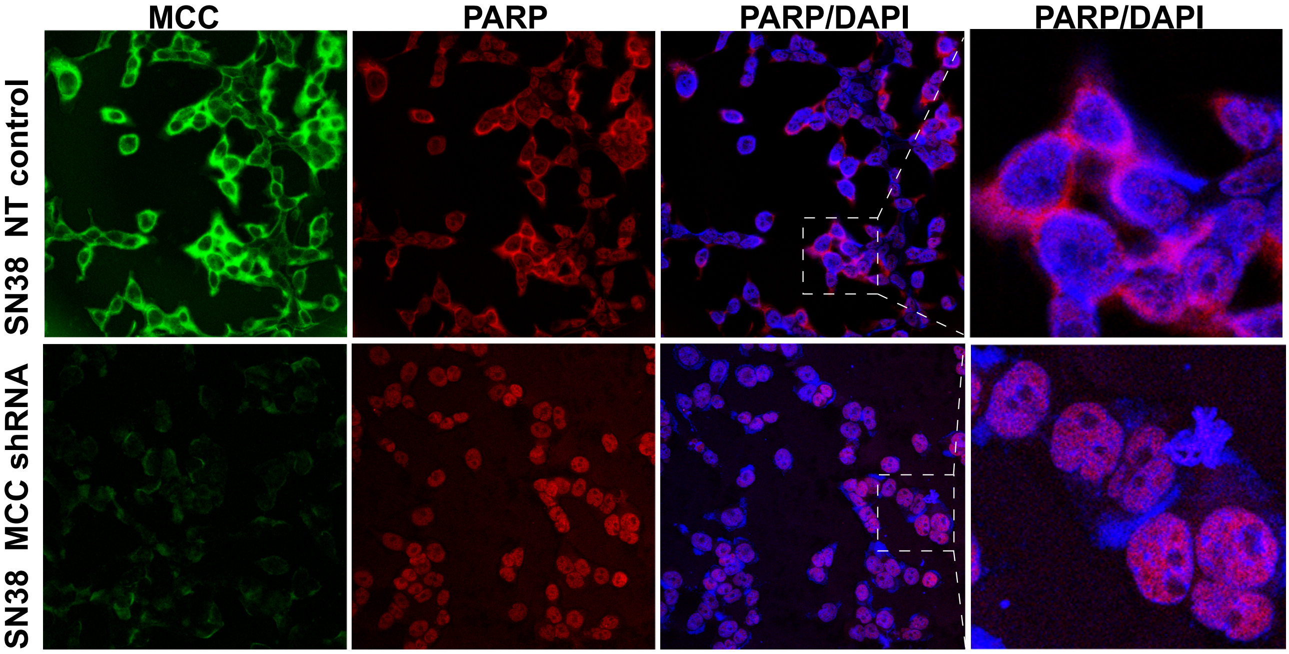

Supplementary Figure S3. PARP subcellular localisation following SN38/irinotecan-induced cytotoxicity in MCC knockdown (MCC shRNA) or non-targeted (NT) HCT116 cells.
HCT116 cells were treated with 1 µM of SN38 for 2 hr. MCC was detected using a Cy3-conjugated anti-mouse antibody (green) and PARP was detected using anti-rabbit Alexa Fluor 647 (far-red) secondary antibody. The nucleus was stained with DAPI and is shown in blue.

Supplementary Table S1. Relative *MCC*-201 mRNA expression levels of 271 TCGA colon cancers and their matching methylation beta-values for 16 CpG sites. Methylation beta-values >0.5 highlighted in orange and beta-values <0.4 highlighted in pink. Green denotes two CpG sites that are mostly hypermethylated in both cancer and normal tissue.

Supplementary Table S2. Relative *MCC*-201 mRNA expression levels of 86 TCGA rectal cancers and their matching methylation beta-values for 16 CpG sites. Methylation beta-values >0.5 highlighted in orange and beta-values <0.4 highlighted in pink. Green denotes two CpG sites that are mostly hypermethylated in both cancer and normal tissue.
