# Supplementary material for: MCC Gene Silencing Is a CpG Island Methylator Phenotype-Associated Factor That Predisposes Colon Cancer Cells to Irinotecan and Olaparib
Source: Cancers (Basel). 2022 Jun 9;14(12):2859. doi: 10.3390/cancers14122859 (PMC9221012; doi:10.3390/cancers14122859)

Western blot images

Jahan et al. *MCC* gene silencing is a CpG island methylator phenotype-associated factor that predisposes colon cancer cells to irinotecan and olaparib. Cancers 2022; 14

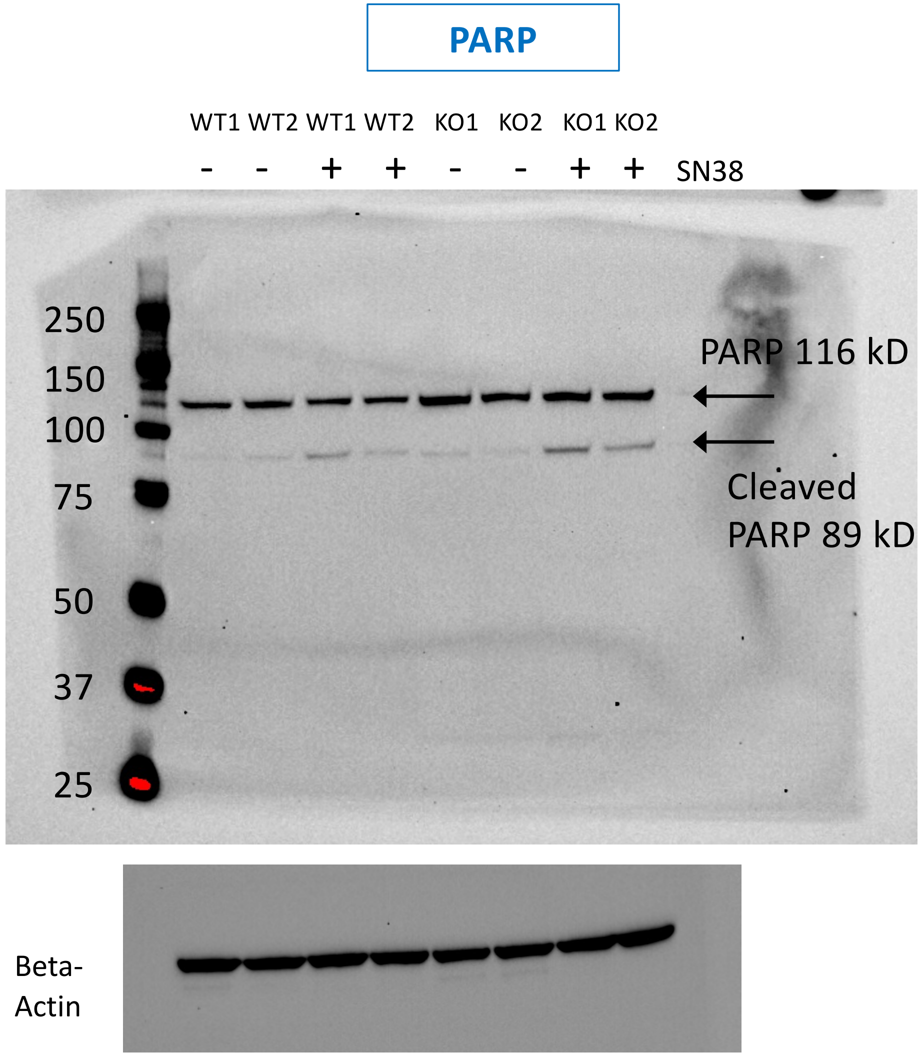
PARP
WT1 WT2 WT1 WT2 KO1 KO2 KO1 KO2
- - + + - - + + SN38
250
150
100
75
50
37
25
PARP 116 kD
Cleaved
PARP 89 kD
Beta-
Actin

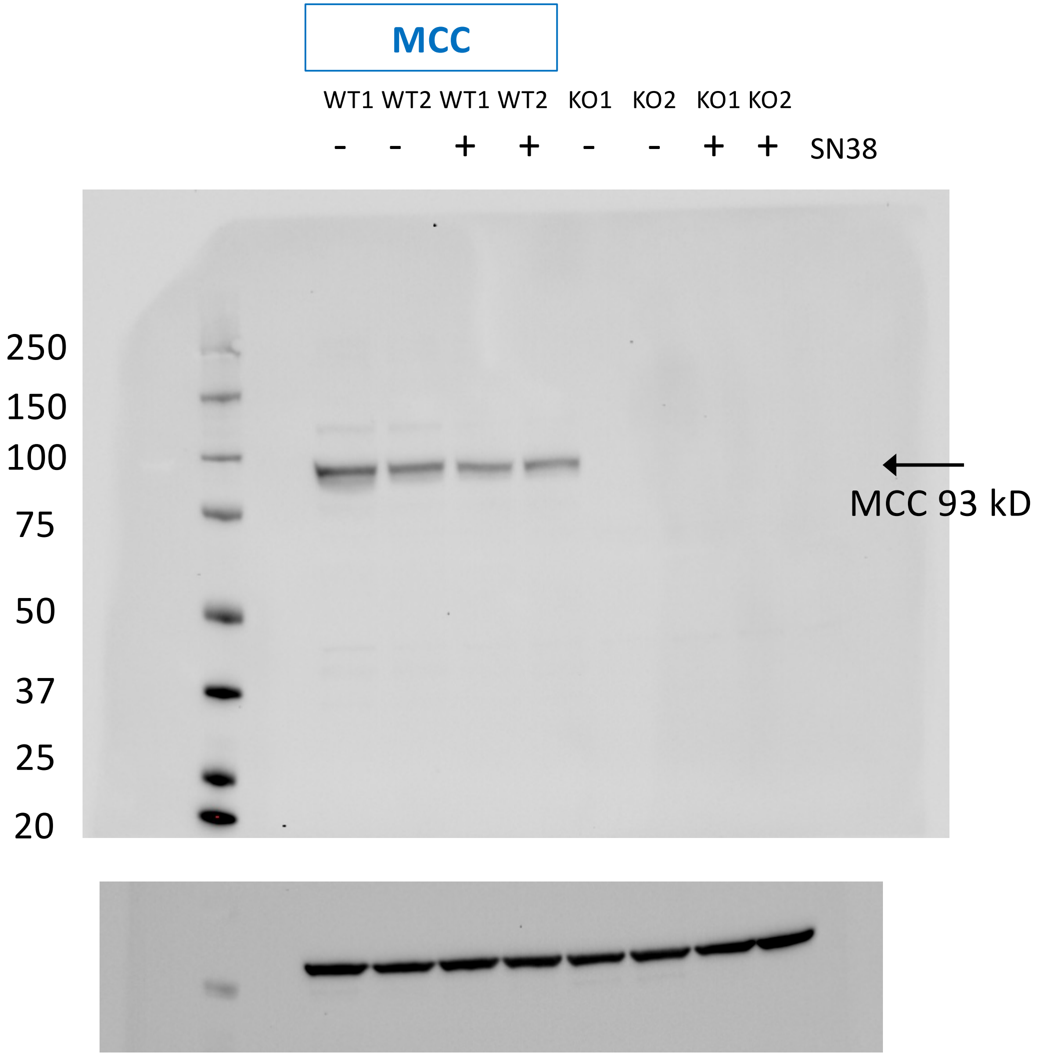
MCC
WT1 WT2 WT1 WT2 KO1 KO2 KO1 KO2
- - + + - - + + SN38
250
150
100
75
50
37
25
20
MCC 93 kD

DNA-PKcs

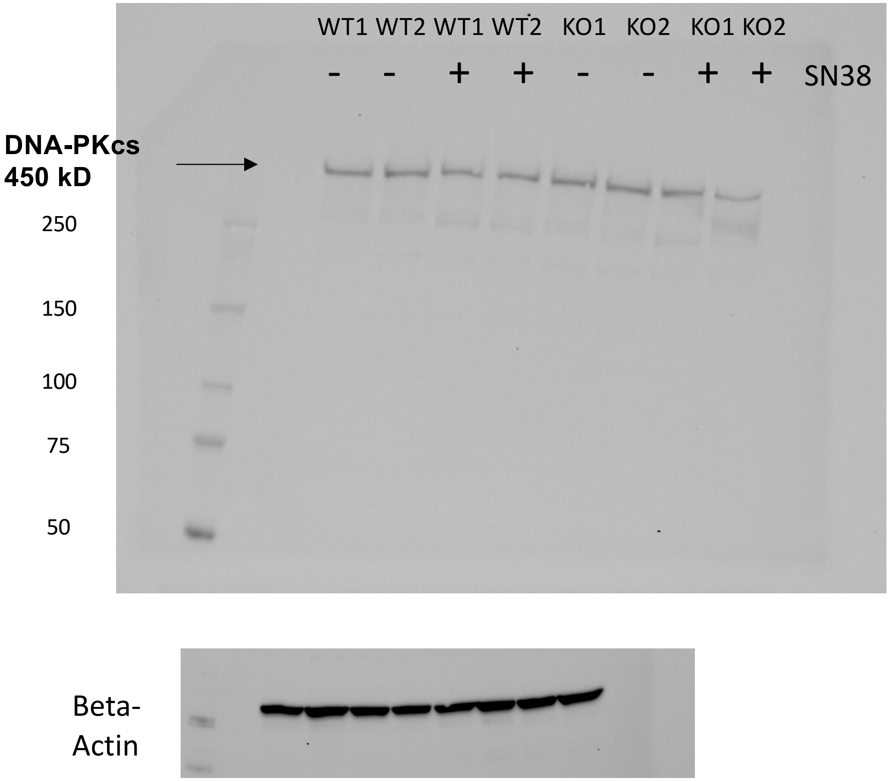

pDNA-PKcs S2056

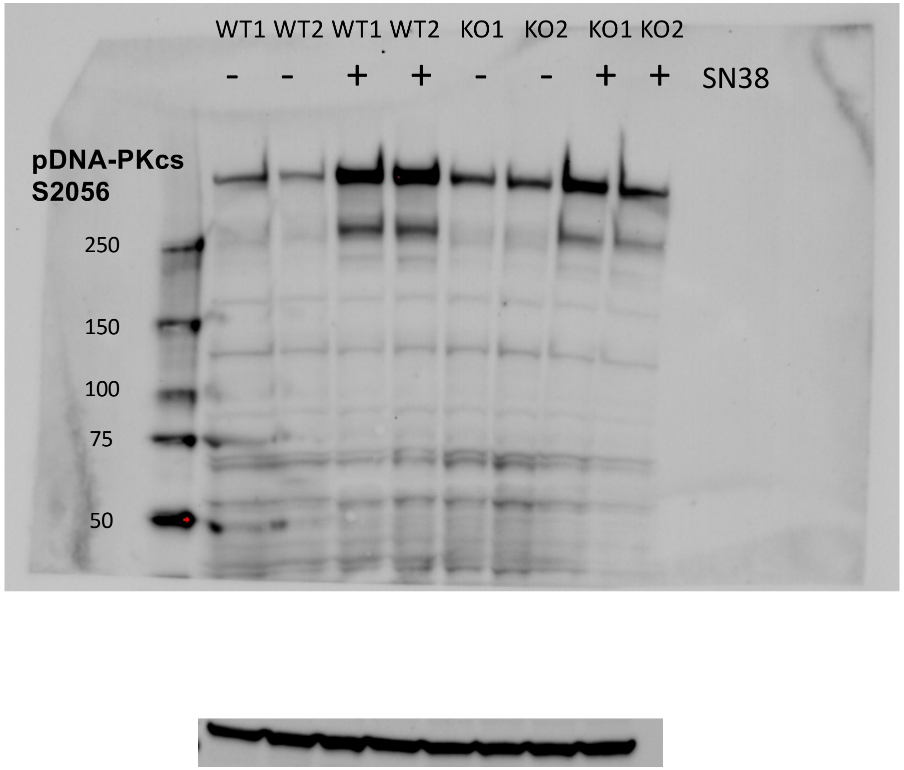

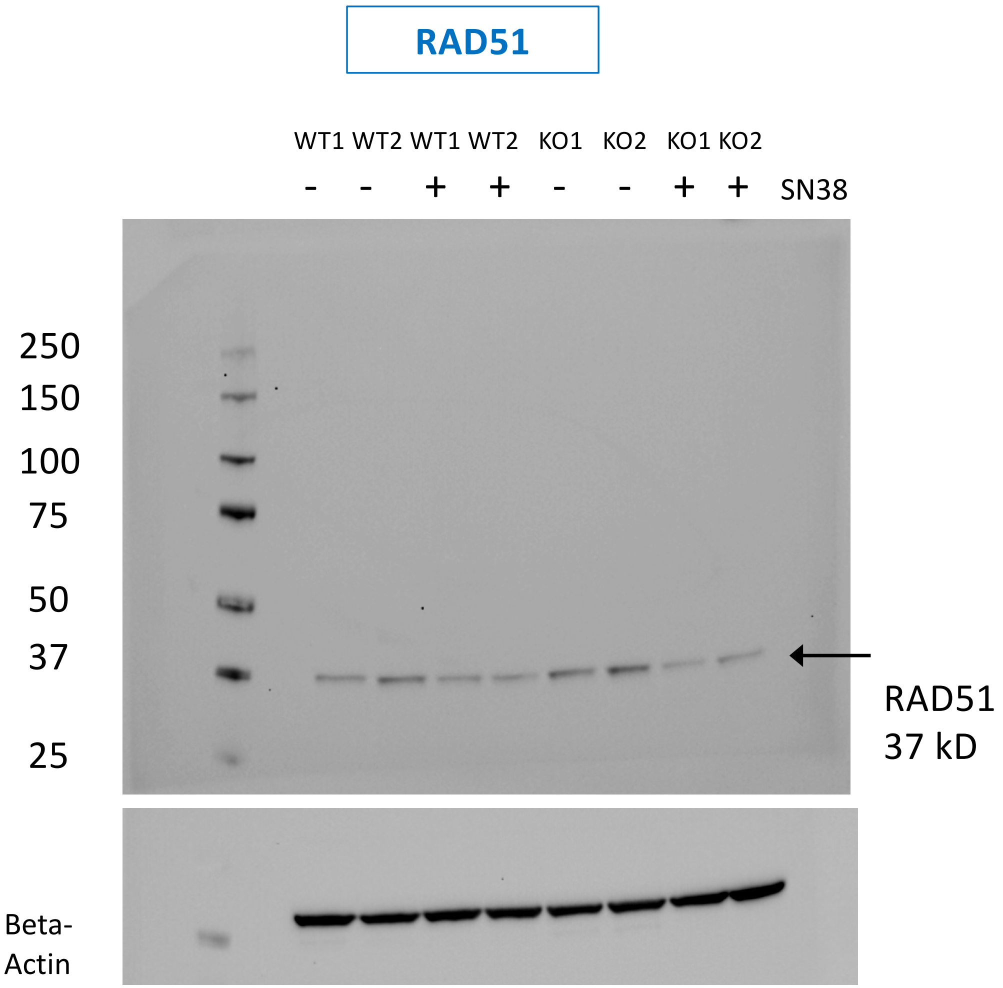
RAD51
WT1 WT2 WT1 WT2 KO1 KO2 KO1 KO2
- - + + - - + + SN38
250
150
100
75
50
37
25
RAD51
37 kD
Beta-
Actin

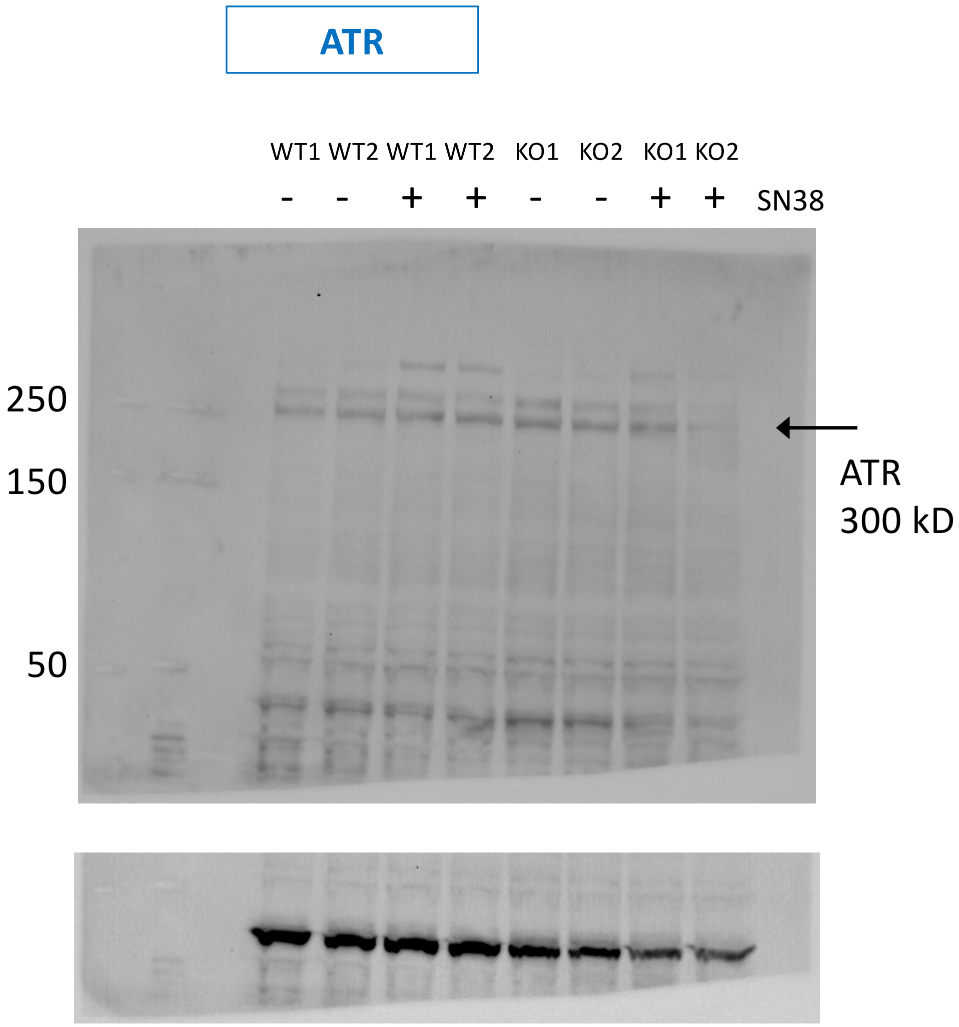
ATR
WT1 WT2 WT1 WT2 KO1 KO2 KO1 KO2
- - + + - - + + SN38
250
150
50
ATR
300 kD

PARP and MCC in HCT116 mouse xenografts

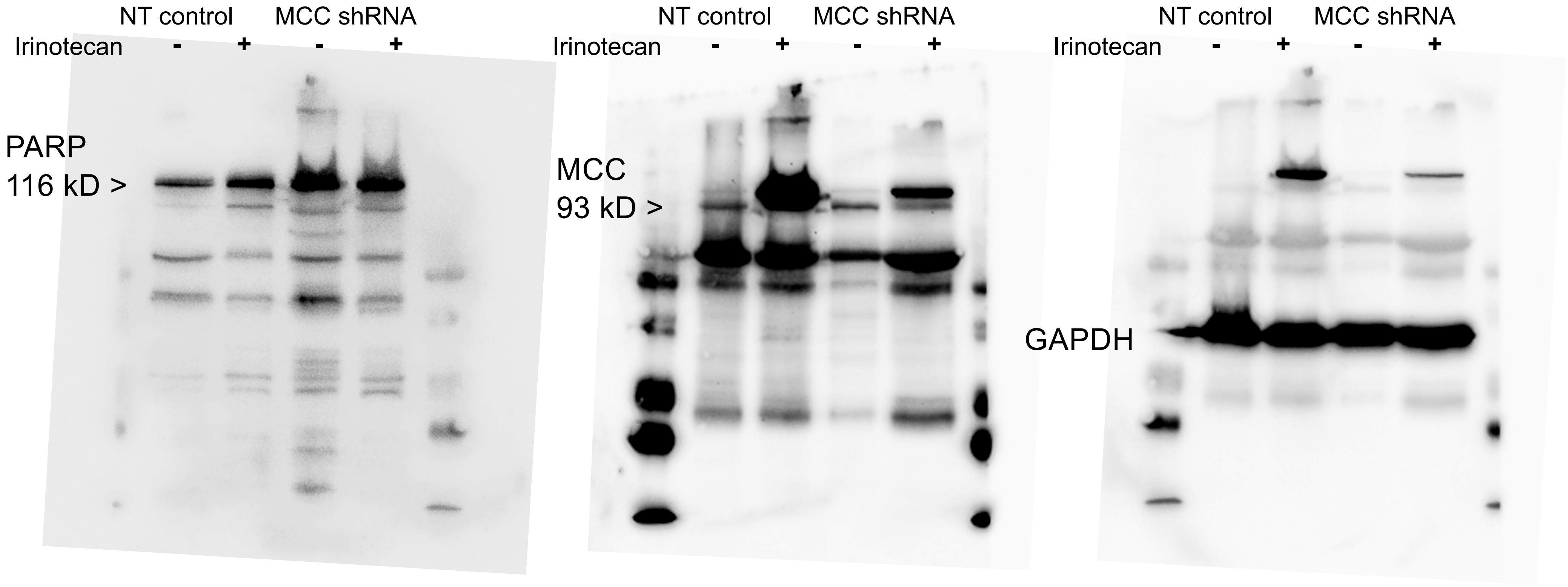

Supplement: Supplementary file 1 [file cancers-14-02859-s001.zip › Figure S4 Western blots.pdf]
